# Fevers

**Published:** 1905-04-08

**Authors:** 


### FEVERS.

**Malaria.**—In opening a discussion on the prophylaxis of malaria at the British Medical Association meeting at Oxford last year, J. W. W. Stephens [1] emphasised several points of importance. All who dwell in the tropics should clearly understand that malaria is a contagious disease, and that the great source of infection lies in the fact that the native population, especially the child population, very frequently carries malarial parasites in its blood without showing the slightest trace of illness. For this reason the segregation of Europeans advocated by Ross, Manson, and others is regarded as most important. The Europeans should congregate in some isolated position half a mile distant from native huts and quarters. Turning to the question of the destruction of *Anopheles*, Stephens points out that about a hundred species of this sub-family exist, but only a dozen or so species are known to convey malaria. Of these *A. maculipennis* has been found to contain the germ in most quarters of the globe. Curiously enough *M. Rossii* appears not to convey the disease. Investigations made in India at Ennur (Madras) and Mian Mir (Punjab) showed the presence at the same time in the same huts of two species of Anophelines, *M. Rossii* and *M. culicifecies*. Dissection of the insects showed in both instances the presence of malarial parasites in a certain number of the latter but not in the former. Hence it is of great practical importance to determine which species in a given district is the medium of infection, so that time, energy and money may not be wasted in destroying insects which, so far as the disease in question is concerned, are harmless. Each species probably has its own particular breeding ground and habits, and it is a matter of great importance that a careful study of the numerous species and their points of difference should be made. The question of the flight of mosquitos also demands attention. Usually apparently they do not travel more than about 200 yards, though exceptionally they may fly a distance of two miles, as appeared to be the case at Mian Mir. At this highly malarial spot the Government of India carried out for two years measures such as the destruction of mosquito larvæ, the filling and draining of wastes and marshes and the destruction of breeding places, with a negative result as regards the diminution of malaria. This seemingly disappointing result should not, however, be permitted to discourage work in a similar direction in view of the brilliant successes obtained at Ismailia and other places. Ross,[2] in a somewhat searching criticism, asserts that the operations were undertaken on far too small and narrow a scale, and over a much too restricted area to give any chance of success. The Mian Mir experiment must therefore be discounted in concerting measures for the prevention of malaria. H. Strachan[3] shows the decrease in malaria which has been obtained in Lagos, West Africa, during the five years 1899-1903 as a result to the prophylactic measures taken. The number of cases, notwithstanding an increasing population, native and European, fell from 1594 in 1899 to 970 in 1903. Deaths during the same period fell from 624 to 427. Care has been taken to teach the natives the cause and prevention of malaria, and the ordinary prophylactic measures have been employed, such as destruction of anopheles breeding grounds, prophylaxis by quinine, the use of mosquito nets, etc. The mosquito proof room, though effective, is acknowledged to be unbearably hot as ordinarily constructed. Strachan recommends, as cheap and portable, a large umbrella of waterproof canvas with cane ribs and bamboo support. Over the umbrella is laid a circular piece of calico to the edges of which are attached an ample mosquito net weighted and reaching to the ground. There is room for a table and chair inside, so that writing, reading, and dining can be pursued in some comfort. Usually the value of quinine prophylaxis is indisputable, but it is not invariable. In some earlier military expeditions it failed, and recently F. Beringer[4] found it ineffectual in Hong Kong. Predisposing causes of malaria also should be considered. R. Cadwallader[5] considers hydræmia is an important factor in liability to infection. J. L. Powell[6] found species of *Culex* but no *Anopheles*, larvæ or insects, in the neighbourhood of Fort Hamilton, New York, when pursuing his researches into malaria there. A. Duncan,[7] speaking of prophylaxis for armies on the march, draws attention to the importance of general measures, such as good food, dry sleeping places, woollen clothing, avoidance of overfatigue, and especially of boiling all water used for drinking purposes in reducing susceptibility to infection.

[1] Brit. Med. Jour., Sept. 17. [2] Ibid. [3] Ibid. [4] Ibid. [5] Med. Record, Sept. 17. [6] Ibid., Nov. 19. [7] Brit. Med. Jour., Sept. 17.

*(To be continued.)*

